# Effect of partially replacing soybean meal with sunflower meal with supplementation of multienzymes on growth performance, carcass characteristics, meat quality, ileal digestibility, digestive enzyme activity and caecal microbiota in broilers

**DOI:** 10.5713/ab.21.0553

**Published:** 2022-03-03

**Authors:** Muhammad Umar Yaqoob, Muhammad Yousaf, Safdar Imran, Safdar Hassan, Waqar Iqbal, Muhammad Umer Zahid, Naveed Ahmad, Minqi Wang

**Affiliations:** 1 College of Animal Science, Zhejiang University, Key Laboratory of Animal Nutrition and Feed Science (Eastern China), Ministry of Agriculture, Hangzhou 310058, China; 2 Institute of Animal and Dairy Sciences, University of Agriculture, Faisalabad 38000, Pakistan; 3 Department of Animal Nutrition, University of Veterinary and Animal Science, Lahore 54000, Pakistans

**Keywords:** Broiler, Cecal Microflora, Intestinal Morphology, Multi-enzymes, Nutrient Digestibility, Sunflower

## Abstract

**Objective:**

An experiment was conducted to evaluate the effects of partially replacing soybean meal (SBM) with sunflower meal (SFM) with added exogenous multienzymes (MEs) on various biological parameters in broilers.

**Methods:**

One week-old, 400 broiler chicks were randomly divided into four treatments (control, 3SFM, 6SFM, and 9SFM) with 5 replicates/treatment (20 chicks/replicate). Control diet was without SFM and MEs, while diets of 3SFM, 6SFM, and 9SFM treatments were prepared by replacing SBM with SFM at levels of 3%, 6%, and 9%, respectively, and were supplemented with MEs (100 mg/kg). Feeding trial was divided into grower (8 to 21 day) and finisher phases (22 to 35 day). External marker method was used to measure the nutrient digestibility. At the end of trial, twenty birds (one birds per replicate) with similar body weight were slaughtered for samples collection.

**Results:**

No significant effect of dietary treatments was found on all parameters of growth performance and carcass characteristics, except relative weight of bursa. Weight (25.0 g) and length (15.80 cm) of duodenum were significantly (p<0.05) higher in 3SFM than control. Lowest (p<0.05) villus height/crypt depth ratio was found in 3SFM and 9SFM than control. Most of meat quality parameters remained unaffected, however, highest pH of breast meat (6.16) and thigh meat (6.44) were observed in 9SFM and 3SFM, respectively. Lowest (p<0.05) cook loss of thigh meat was found in 6SFM (31.76%). Ileal digestibility of crude protein was significantly (p<0.05) higher in 3SFM (72.35%) than control (69.46%). In addition, amylase (16.87 U/mg) and protease (85.18 U/mg) activities were significantly (p<0.05) higher in 3SFM than control. However, cecal microbial count remained unaffected.

**Conclusion:**

Partial replacement (up to 9%) of SBM with SFM, with added MEs can help to improve the nutrient digestibility, intestinal morphology, and digestive enzyme activities without affecting cecal microbial count and growth performance in broilers.

## INTRODUCTION

Feed contributes more than 70% of total cost of production in commercial poultry industry and increasing cost of production demands the use of cheaper and efficient ingredients for balanced feed formulation. The availability of protein sources is becoming limited overtime across the world and protein is one of the most expensive nutrients in poultry diets [1,2]. Soybean meal (SBM) is an important protein source used in poultry diets [3]. Yet, due to increasing prices, alternative feed ingredients are required for formulation of a balanced and economical diet [4].

Sunflower meal (SFM), a byproduct obtained after oil extraction from sunflower seeds. The SFM is relatively cheaper source of protein and serves as suitable alternative to SBM in poultry feed formulation [5], because of its good protein level, high contents of calcium, phosphorus, vitamin B-complex, and low levels of antinutritional and toxic compounds [6]. The crude protein (CP) level of SFM varies from 29% to 45%, depending upon the oil extraction and dehulling process and remains inverse to fiber contents [7,8]. Yet, high levels of insoluble fiber and low levels of essential amino acids (lysine and methionine), are limiting factors for the use of SFM in poultry diets [3]. However, high fiber contents in SFM indirectly favors the environment because they are used by bacteria to produce volatile fatty acids which decreases pH of the manure [9] and may be helpful in lowering blood cholesterol levels [3]. In addition, increasing levels of SFM has been reported to decrease the liver fat contents [2,10].

Though, high fiber level in SFM is resistant to the bacterial dilapidation in gastrointestinal tract (GIT) which can either be overcome by reducing the fiber contents of SFM by heat treatment, pelleting the feed or by supplementing the feed with enzymes suitable to digest SFM non-starch polysaccharides [3]. The SFM fed alone can cause problems for the ileum mucosal cells leading to reduced nutrients absorption [11], however addition of exogenous enzymes has positive impacts in diets containing SFM. The SFM can be included in diet at varying levels despite there being deficiency of limiting amino acids [12]. Use of SFM with supplementation of exogenous enzymes had no negative effect on growth performance in broilers [13]. Similarly, improvement in CP and fiber digestibilities was observed by using SFM with enzymes [14]. The current study was planned with objectives to explore the effects of partially replacing SBM with SFM in diet with supplementation of multienzymes (MEs) on growth, meat quality and other biological parameters in the broilers.

## MATERIALS AND METHODS

### Animals and experimental design

The protocol of this study was approved (IADS/733) for ethical use, care, and welfare of experimental animals by Institute of Animal and Dairy Sciences, University of Agriculture, Faisalabad, Pakistan.

Experiment was conducted on 400 Arbor Acres broiler chicks (both male and female) at Research and development farm of Five Star Feeds Pvt. Ltd., Gujranwala, Pakistan. After one week of feeding on commercial starter diet (CP 23 %; metabolizable energy 3,000 kcal/kg), the chicks were randomly divided into four treatments with five replicates per treatment and 20 chicks per replicate. T1: control, without SFM and MEs; T2: 3SFM, 3% SBM was replaced with SFM; T3: 6SFM, 6% SBM was replaced with SFM; T4: 9SFM, 9% SBM was replaced with SFM. Diets of T2, T3, and T4 were supplemented with MEs (100 mg/kg). Axtra XAP 101 (Danisco Animal Nutrition, Marlborough, UK) was used as a source of enzymes with composition of 1,4-β-xylanase 20,000 U/g: α-amylase 2,000 U/g and protease 40,000 U/g. The formal feeding trial was divided in to two phases, (grower phase 8 to 21days and finisher phase 22 to 35 day). Diets of both phases were formulated according to breed specific guide (Arbor Acres broiler nutrition specification, 2019; Table 1 and 2). Birds were provided with free access to feed and water throughout the experiment.

### Samples collection and measurements

#### Growth performance

Data regarding feed intake and body weight were recorded on weekly basis. The collected data were arranged to calculate average weight gain, feed intake and feed conversion ratio (FCR) during grower and finisher phases and on overall basis.

#### Carcass characteristics

At the end of feeding trial, twenty birds (one birds per replicate) with similar body weight were slaughtered to measure carcass traits. Live body weight, carcass weight and weight of different body parts (breast, thigh, drumstick, wings, and abdominal fat) and internal organs (heart, liver, gizzard, bursa, spleen, and thymus) was recorded. Dressing percentage was calculated by dividing carcass weight to live body weight. Body parts and organs weight was expressed as a percent of carcass weight. The weight and length of the intestinal segments (duodenum, jejunum, ileum, and ceca) were also measured and recorded.

#### Meat quality analysis

The water holding capacity (WHC) of breast and thigh meat samples was determined by method of Jang et al [15]. For determination of WHC, the meat samples were centrifuged (Eppendorf Centrifuge 5804R, Taufkirchen, Germany) at 5,000 rpm for 10 minutes at 4°C. The cooking loss was determined by cooking meat samples (breast and thigh) at 90°C for 30 minutes in hot water bath followed by cooling at room temperature. The sample weight before (W_1_) and after (W_2_) cooking was recorded, to calculate the cooking loss using following formula:


$$Cooking\;loss\;(\%)=\frac{\left({\text{W}}_{1}-W_{2}\right)}{{\text{W}}_{1}}\times 100$$

Penetrometry of breast muscles were done to determine the shear force using texture analyzer (Texture Analyzer TX-700; LAMY Rheology, Champagne au Mont d’Or, France) in triplicates. Cooked breast muscle fibers were kept perpendicular to the blade and analyzer was used with following operational parameters: maximum speed: 1 mm/s; measure time: 10 seconds; force to start: 2 g; wait position: 10 mm; force set: 1,000 g and maximum distance 50 mm. To determine the pH value of meat sample (breast and thigh) at 24 h, slurry was prepared by homogenizing (OV5; VELP Scientifica, Usmate Velate, Italy) the meat sample (10 g) in distilled water (90 mL) and pH was recorded in duplicate using pH meter (HI 99163; Hanna Instruments Inc., Woonsocket, RI, USA). Meat colorimeter (STPR45; Precision colorimeter, Shenzhen, China) was used to determine the color of breast and thigh meat samples in triplicates. Calibration was done with black and white calibration tiles before using the apparatus according to the manufacturer instructions and CIE L, a, and b values were recorded.

#### Digestibility trial

The nutrient digestibility was determined by external marker method [16]. External marker Celite (1%) was added in the last five-day feeds. Ileal contents were collected from slaughtered birds and composited for each group and stored at −10°C till further analysis. Proximate analysis for determination of dry matter (DM), CP, ether extract (EE), and crude fiber (CF) of feed and ileal contents were done following A.O.A.C. (2000). Following equation was used for calculation of digestibility coefficient:


$$Digestibility\;coefficient\;(\%)=100-(100\times \frac{Marker\;in\;feed\;(\%)}{Marker\;in\;ileal\;contents\;(\%)}\times \frac{Nutrient\;in\;ileal\;contents\;(\%)}{Nutrient\;in\;feed\;(\%)})$$

#### Digestive enzymes activity

Jejunal digesta was collected from the slaughtered birds and diluted by 4× and 10× with phosphate-saline buffer followed by centrifugation at 3,000×g for 15 minutes and 18,000×g for 20 minutes at 4°C, respectively. The supernatant was collected and stored at −70°C till further analysis. Activities of lipase, amylase and protease were determined using corresponding diagnostic kits according to manufacturer’s instructions (Shanghai Changjin Biotechnology Co., Ltd., Shanghai, China). The method of Lowry et al [17] was followed for protein measurement, using bovine serum albumin as standard.

#### Jejunal morphology

The intestinal segment (jejunum) was dehydrated and embedded in paraffin for slides preparation by sectioning with microtome (5 μm) and slides were stained using hematoxylin and eosin. Morphometric analysis was done by following the previous method [18] with the help of light microscope having computer-assisted morphometric system (Nikon Corporation, Tokyo, Japan).

#### Quantification of cecal microflora

Cecal contents were also collected from slaughtered birds to determine the quantity of different bacteria. Samples were collected and homogenized followed by dilution with sterile 0.9% saline (1:4 w/v). Agar plate culture technique was followed for quantification of bacteria. Tenfold dilution of each sample was done and placed on three replicate plates: for *Lactobacillus* in modified Rogosa, *Bifidobacterium* in TOS Propionate agar, *Escherichia coli* (*E. coli*) in MacConkey agar and *Clostridium perfringens* (*C. perfringens*) in tryptose sulphite cycloserine agar. Plates were incubated under anaerobic conditions (except MacConkey agar plates) for 24 to 48 h at 37°C. Following incubation, the bacteria colonies were counted and reported as the log of colony-forming unit (CFU) per gram of digesta.

### Statistical analysis

Data were expressed as the mean±standard error of the mean. All data collected were subjected to one-way analysis of variance using general linear model procedure in SPSS 26. Dietary treatments were used as independent variables and replicates under each treatment were served as statistical unit. Statistical difference among the means were determined through Tukey’s test (p<0.05).

## RESULTS

### Growth performance

No significant effect (p>0.05) of dietary treatments was found on growth performance in broilers throughout the feeding trial (Table 3). During grower phase (8 to 21 days), numerically highest feed intake (1,074.82 g) and weight gain (768.94 g) were found in 9SFM group however, FCR of control group (1.33) was better than that of other treatments but difference was non-significant (p>0.05). During finisher phase (21 to 35 days), highest feed intake (2,178.96 g) and weight gain (1,272.41 g) were found in control and 6SFM, respectively. Overall data showed highest weight gain (1,999.51 g) and better FCR (1.56) of 3SFM, but difference was non-significant (p>0.05) with other groups.

### Carcass characteristics

Results exhibited no significant (p>0.05) effect of dietary treatments on different carcass parts (Table 4). Numerically highest (68.13%) dressing percentage was found in control followed by 3SFM (67.26%). On the other hand, highest (p>0.05) relative breast weight was found in 6SFM (24.87%) and lowest in control (22.25%). Similarly, no significant (p> 0.05) effect of dietary treatments was observed on relative weight of body organs. However, relative weight of bursa was better (p<0.05) in all experimental groups (0.66% to 0.71%) than control (0.45%). While relative weight of other immune organs (spleen and thymus) was not affected by dietary treatments (Table 5).

### Intestinal segments and morphology

Regarding dimensions of different parts of intestine, weight of duodenum was improved by replacing SBM with SFM with supplementation of MEs. Highest (p<0.05) weight of duodenum (25.0 g) was observed in 3SFM and lowest in control (19.6 g). In addition, length of duodenum (15.8 cm) and jejunum (77.3 cm) were also significantly (p<0.05) higher in 3SFM than control (Table 6). While length and weight of remaining parts were not affected by dietary treatments. Results of jejunal morphology showed highest villus height (VH) (1,221.60 μm) in 3SFM followed by control (1,187.60 μm) and 6SFM (1,027.80 μm). There was non-significant (p>0.05) difference for VH among control, 3SFM, and 6SFM. Significantly lowest VH/crypt depth (CD) ratio was found in 9SFM (4.97) and highest in control (5.86). The VH/CD ratio in 3SFM and 9SFM were statistically similar (Table 6).

### Meat quality analysis

Most of the meat quality parameters remained unaffected by dietary treatments (Table 7). pH of muscles (breast and thigh) was increased by replacing SBM with SFM and supplementation of MEs. Highest (p<0.05) pH of breast meat was found in 9SFM (6.16) and lowest on control (5.90). pH of thigh meat was highest in 3SFM (6.44) which was statistically similar to other experimental groups but higher than control. Lowest (p<0.05) cook loss of thigh meat was found in 6SFM (31.76%), followed by control (31.91%) and highest in 3SFM (35.62%). In addition, breast meat color (b*, yellowness) was also affected by dietary treatments. Value of b* was similar in Control and 6SFM (13.69) but low in 3SFM (10.57) and 9SFM (10.41).

### Ileal digestibility

Results showed no significant effect of dietary treatments on ileal digestibility of DM, EE, and CF however, CP digestibility was significantly improved (Table 8). Highest ileal digestibility of CP was found in 3SFM (72.35%) followed by 6SFM (71.79%), digestibility values of these two groups were statistically similar and higher than control (69.46%). Similarly, numerically highest (p>0.05) ileal digestibility of EE (75.11%) and CF (17.99%) were found in 3SFM.

### Digestive enzymes activity

Enzymatic activities of lipase, amylase, and protease in jejunal digesta is presented in Table 9. The amylase (16.87 U/mg) and protease (85.18 U/mg) activities were significantly (p< 0.05) higher in 3SFM than Control. However, no significant difference was observed for amylase activities within the experimental groups. While lipase activities were not affected by dietary treatments (p>0.05).

### Quantification of cecal microflora

No significant (p>0.05) effect of dietary treatments was found on cecal microbial counts (Table 10). However, highest counts of *Lactobacillus* spp. (11.0 log CFU/g) and *Bifidobacterium* spp. (9.80 log CFU/g) were found in 6SFM. On the other hand, highest counts of *Clostridium perfringen* (6.40 log CFU/g) and *E. coli* (6.60 log CFU/g) were found in control.

## DISCUSSION

Several factors and problems such as intense global competition between producing countries, permanent changes in social, political and consumer perceptions regarding food safety, animal welfare and environmental protection are influencing poultry production and health [19]. Additionally, the recent pandemic coronavirus disease of 2019 (COVID-19) tremendously influenced the sustainability of poultry production. It caused severe damage to the marketing and distribution networks where farmers have failed to receive necessary supplies across the borders [20]. Keeping in view of this scenario the present study was planned to evaluate the effect of partial replacement of SBM with SFM with added MEs on different biological parameters in broilers. The SFM can partially replace SBM with added MEs without any adverse effect on the feed intake, weight gain and FCR. Similar results were reported by Alagawany et al [21] for feed intake and feed efficiency in broilers. Oliveira et al [22] reported no difference in performance of broilers during early days of age (1 to 21 d) and entire rearing experiment (1 to 42 d), and reported that up to 10% inclusion of SFM during 21 to 42 days of age is safe in broilers [23]. Likewise, Horvatovic et al [24] stated that use of SFM in broiler’s diet did not affect the feed intake. During grower phase FCR was better in Control while during finisher phase FCR in 3SFM was better than other treatments however, difference among them was non-significant (p>0.05). Similarly, overall data of growth performance showed non-significant (p>0.05) effect of dietary treatments on FCR. Previous study stated that use of SFM up to 20% in broiler had no effect on FCR in broilers [25]. While other results suggested that, SFM up to 20% and even higher level had no adverse impact on weight gain or live weight in broilers [10, 26,27]. Contrary, another study reported worsening performance of broilers on addition of SFM in diet [25]. Conflicting results might be due to use of higher replacement levels of SFM. As SFM has high levels of fiber which might be the reason of negative effects on different parameters of growth performance in broilers. Even controversy exists in the use of enzymes combinations, which also affected the growth of broilers differently.

Like previous studies, use of SFM with added MEs did not affect carcass parameters in broilers [27]. Similarly, carcass yield was not affected by using SFM in broiler’s diet [24]. Yet, improvement in carcass percentage with supplementation of enzymes at high SFM inclusion was reported by Omojola and Adesehinwa [28]. Contrary to these results, negative effect of SFM inclusion was found in carcass parameters of broiler [29]. There was no effect of low level of SFM inclusion while higher level depresses the carcass traits [10]. There was no significant effect of SFM inclusion in broiler diet on liver, abdominal fat, carcass, dressing, gizzard, and goblet [30]. Relative weight of bursal was significantly high in all experimental groups than control. The GIT presented variation in response to changing dietary ingredients in broilers. The weight of duodenum was significantly highest in 3SFM and decreases with increasing SFM inclusion, and lowest was in Control. Similarly, weight of small intestine was significantly increased by replacing SBM with SFM in broiler’s diet [31]. In addition, duodenum and jejunum length was also enhanced by using SFM with MEs in diets. The length of the intestine was not influenced by the SFM inclusion level while intestinal weight was significantly different on total replacement of SBM with SFM [10]. Contrary result from different studies might be due to using different dietary levels of SFM, with or without MEs or even different combination of MEs. The VH reaches its maximum size in 6 to 8 days post hatching in duodenum and 10 days in ileum and jejunum [32]. The jejunal VH in broilers was higher at 7% SFM and the lowest in 21% SFM in diet, the increasing dietary SFM level resulted in decreasing VH and increasing CD in duodenum and jejunum [7]. Berwanger et al [23] reported decrease in jejunal VH, increase in cryptic depth and decrease in villus cryptic ratio in response to increasing sunflower cake in broiler diet. Similar to our results, jejunal VH was higher in birds fed on MEs supplemented SFM and a linear decrease in the jejunal VH and CD with increasing SFM level occurred [22]. The CD was significantly (p<0.05) higher in 3SFM than all other groups while minimum was in control. The ratio of VH:CD in jejunum was 5.44 at 14% SFM and 3.04 at 21% SFM, presenting quadratic response to SFM [8]. The VH:CD ratio was the highest in control and the lowest in 9SFM in present study. VH:CD ratios were statistically similar in 3SFM and 9SFM groups so, SBM replacement with SFM (with MEs) significantly decreased the VH:CD ratio in broilers. The intestinal morphology impairment due to SFM inclusion in diet can be attributed to cell extrusion rate, high fiber contents in intestine which affect the intestinal epithelium and mucin layer [33] as high fiber contents have abrasive effect on intestinal wall, in particular on villus apex [34]. The elevation in nutrient absorption is associated with the increase in villus surface area and the VH. The higher VH and VH:CD ratios work as indicators of better health of the gut, maturity, and function of mucosa. The decreased VH and increased CD decreases the nutrient absorption surface area resulting in poor absorption, increased secretion, and decreased efficiency of feed [7].

Meat color, pH and texture has a significant correlation indicating the functionality of meat characteristics. Color of the meat affect the acceptability by consumer, so it is an important quality parameter [5]. Different factors including age, sex, pH, pigmentation, processing, and slaughtering conditions affect the meat color. In present study, pH at 24 h for breast and thigh meat in control was significantly (p<0.05) lower. The significant difference in cooking loss may be due to the difference in pH of the meat. The pH value (5.93 to 5.99) of the breast meat after 24 h of slaughtering did not differ significantly in the broilers among experimental groups. The age of slaughtering the bird has impact on the pH of meat, and pH increased by age of broiler [35]. The breast meat cooking loss (%) decreases while WHC (%) increases with increasing dietary SFM, in contrast thigh meat WHC was highest in control and lowest in 9SFM (9% replacement of SBM with SFM+MEs). The color saturation index was significantly higher in the birds fed on the SFM treatment as compared to those fed on SBM, indicating that SFM meal increases the muscle darkness [5].

Similar to the results of Alagawany et al [21], no significant effect of SFM was found on DM and EE digestibility in broilers. Contrary to present study, decrease in DM digestibility was observed by using SFM in diet [10], which might be due to using SFM without enzyme supplementation, leading to increased fiber contents and viscosity. The CP digestibility was significantly (p<0.05) improved by replacing SBM with SFM at the levels 3% to 6% (with MEs supplementation). These results are also supported by previous study, that enzyme supplemented SFM diet increases protein digestibility [14]. The CF digestibility was not affected significantly (p>0.05) by dietary treatments however, numerical values of CF digestibility of MEs supplemented SFM diets were higher than Control. Addition of exogenous enzymes helped in decreased viscosity and ultimately better absorption; the results are in line with Alagawany et al [21] that addition of exogenous enzyme resulted in better digestibility coefficient than non-supplemented treatments. The digestibility of SFM is affected by many factors including enzyme addition, fiber contents, pelleting method and other factors that need to be explored [3]. Digestive enzymes activities vary in response to variation in diet composition in broilers. Decrease in protease activity was observed by increasing SFM (3% to 9%) in diet (with supplementation of MEs) which was consistent with previous results [7]. This decrease in protease activity may be attributed to presence of chlorogenic acid, an inhibitor. In contrast, dietary SFM (7, 14 and 21%) did not affect the ileal protease activity [7] in broilers. While decrease in ileal protease activity on 75% SBM dietary substitution with SFM was observed in broilers [30]. Contrary to present study, the protease, and α-amylase activities in chick digesta were not significantly affected, when fed on SFM based diet [7]. Alagawany et al [30] found increase in ileal amylase activity in broilers fed on sunflower seeds and also reported that enzyme addition and SFM interaction is important for ileal enzyme activity in broilers.

Dietary fibers affect the composition and growth of microbes in the gut as soluble fiber may act as prebiotics and insoluble as nutrient diluent. Present study exhibited that partial replacement of SBM with SFM with supplemented MEs has no effect on cecal bacterial counts, which might be due to very low levels of fiber present in SFM used in replacement of SBM or added MEs degrade them and might be the reason of no effect of using SFM on microbial counts. On the other hand, Mikulski et al [36] stated that SFM (21%) significantly increased cecal *E. coli* counts as compared to Control in turkeys, which might be due to very high levels of SFM used or that no enzyme supplementation was done. The dietary fiber improved gut health in poultry by preventing pathogenic microbial adhesion to epithelial mucosa. Similarly, reduction in *E. coli* population and increase in *Lactobacillus* population was also observed by Abazari et al [37] in cecal contents of broilers fed on rice husk.

## CONCLUSION

Partial replacement (up to 9%) of SBM with SFM with supplementation of MEs in broiler’s diets can help to improve digestive enzymes activity leading to improved nutrient digestibility and intestinal morphology, without any effect on growth performance, meat quality and cecal bacterial counts.

## Figures and Tables

**Table 1:** Ingredients and nutrients composition of grower diets (8 to 21 days)

Items	Treatments^1)^			

	Control	3SFM	6SFM	9SFM
Ingredients (%)				
Maize	55.67	52.98	48.99	45.01
Soya bean meal	27.79	24.75	21.79	18.79
Sunflower meal	0.00	2.75	5.71	8.71
Rapeseed meal	8.00	10.74	13.71	16.64
Veg. oil	5.04	5.30	6.40	7.50
Limestone	1.24	1.19	1.15	1.10
Monocalcium phosphate	0.74	0.74	0.73	0.72
L-Lysine HCl	0.38	0.42	0.44	0.46
DL-Methionine	0.27	0.25	0.22	0.20
Threonine	0.08	0.08	0.07	0.07
NSPase	0.00	0.01	0.01	0.01
NaCl	0.25	0.25	0.24	0.24
Sodium bicarbonate	0.23	0.23	0.23	0.24
Phytase	0.01	0.01	0.01	0.01
VitaMin premix^2)^	0.30	0.30	0.30	0.30
Total	100.00	100.00	100.00	100.00
Metabolizable energy (Kcal/kg)	3,100			
Crude protein	21.50			
Lysine	1.29			
Methionine	0.51			
Calcium	0.87			
Available P	0.44			

1) Control: without sunflower meal and multienzymes; soybean meal was replaced with sunflower meal at levels of 3%, 6%, and 9% in 3SFM, 6SFM, and 9SFM, respectively, all diets were supplemented with 100 mg multienzymes per kg diet. Axtra XAP-101 (1,4-β-xylanase 20,000 U/g; α-amylase 2,000 U/g; Protease 40,000 U/g, Danisco, UK) was used as a source of multienzymes.

2) Vit. A 15,000 IU/kg, D3 3,000 IU/kg, E 60 IU/kg, K_3_ 3 mg/kg, B_1_ 2 mg/kg, B_2_ 8 mg/kg, niacin 45 mg/kg, pantothenic acid 15 mg/kg, B_6_ 4 mg/kg, folic acid 1 mg/kg, B_12_ 1 mg/kg, choline chloride (60%) 500 mg/kg, magnesium sulphate 53 mg/kg, manganese sulphate 18.5 mg/kg, zinc sulphate 2 mg/kg, ferrous sulphate 35 mg/kg and copper sulphate 45 mg/kg.

**Table 2:** Ingredients and nutrients composition of finisher diets (22 to 35 days)

Items	Treatments^1)^			

	Control	3SFM	6SFM	9SFM
Ingredients (%)				
Corn	62.27	59.79	56.39	52.98
Soya bean meal	23.56	20.56	17.56	14.56
Sunflower meal	0.00	2.94	5.94	8.94
Rapeseed meal	6.09	8.49	11.00	13.52
Veg. oil	5.18	5.35	6.30	7.24
Limestone	1.03	0.99	0.95	0.91
Monocalcium phosphate	0.51	0.50	0.50	0.49
L-Lysine HCl	0.34	0.36	0.38	0.40
DL-Methionine	0.23	0.21	0.19	0.17
Threonine	0.06	0.06	0.05	0.06
NSPase	0.00	0.01	0.01	0.01
NaCl	0.22	0.22	0.21	0.21
Sodium bicarbonate	0.20	0.21	0.21	0.20
Phytase	0.01	0.01	0.01	0.01
VitaMin premix^2)^	0.30	0.30	0.30	0.30
Total	100.00	100.00	100.00	100.00
Metabolizable energy (Kcal/kg)	3,200			
Crude protein	19.50			
Lysine	1.16			
Methionine	0.47			
Calcium	0.79			
Available P	0.40			

1) Control: without sunflower meal and multienzymes; soybean meal was replaced with sunflower meal at levels of 3%, 6%, and 9% in 3SFM, 6SFM, and 9SFM, respectively, all diets were supplemented with 100 mg multienzymes per kg diet. Axtra XAP-101(1,4-β-xylanase 20,000 U/g; α-amylase 2,000 U/g; Protease 40,000 U/g, Danisco, UK) was used as a source of multienzymes.

2) Vit. A 15,000 IU/kg, D_3_ 3,000 IU/kg, E 60 IU/kg, K_3_ 3 mg/kg, B_1_ 2 mg/kg, B_2_ 8 mg/kg, niacin 45 mg/kg, pantothenic acid 15 mg/kg, B_6_ 4 mg/kg, folic acid 1 mg/kg, B_12_ 1 mg/kg, choline chloride (60%) 500 mg/kg, magnesium sulphate 53 mg/kg, manganese sulphate 18.5 mg/kg, zinc sulphate 2 mg/kg, ferrous sulphate 35 mg/kg and copper sulphate 45 mg/kg.

**Table 3:** Effect of partially replacing soybean meal with sunflower meal with added multienzymes on growth performance in broilers

Parameters	Treatments^1)^				p-value

	Control	3SFM	6SFM	9SFM	
Grower (8 to 21 d)					
Feed intake (g)	987.75±51.50	955.27±23.53	964.66±36.07	1,074.82±23.30	0.110
Weight gain (g)	746.57±15.99	710.60±22.73	700.80±37.68	768.94±36.35	0.599
FCR	1.33±0.08	1.35±0.03	1.40±0.06	1.41±0.07	0.743
Finisher (22 to 35 d)					
Feed intake (g)	2,178.96±74.60	2,041.55±34.90	2,170.44±40.60	2,052.81±29.93	0.230
Weight gain (g)	1,159.83±39.35	1,230.57±49.66	1,272.41±30.56	1,188.02±37.06	0.243
FCR	1.89±0.11	1.67±0.06	1.72±0.08	1.73±0.05	0.241
Overall (8 to 35 d)					
Feed intake (g)	3,166.71±56.47	3,116.37±37.82	3,143.62±78.10	3,008.08±32.36	0.459
Weight gain (g)	1,906.40±53.42	1,999.51±44.75	1,973.21±44.23	1,898.62±43.38	0.391
FCR	1.67±0.08	1.56±0.03	1.59±0.04	1.59±0.03	0.490

FCR, feed conversion ratio.

1) Control: without sunflower meal and multienzymes; soybean meal was replaced with sunflower meal at levels of 3%, 6%, and 9% in 3SFM, 6SFM, and 9SFM, respectively, all diets were supplemented with 100 mg multienzymes per kg diet.

**Table 4:** Effect of partially replacing soybean meal with sunflower meal with added multienzymes on carcass parts in broilers

Parameters (%)	Treatments^1)^				p-value

	Control	3SFM	6SFM	9SFM	
Dressing percentage	68.13±0.80	67.26±0.84	65.57±1.57	65.63±1.42	0.335
Breast^2)^	22.25±0.90	23.95±0.24	24.87±1.03	23.99±1.06	0.455
Thigh^2)^	18.01±1.04	18.22±0.84	18.03±0.95	18.68±0.27	0.934
Drumstick^2)^	6.09±0.25	6.41±0.20	6.51±0.18	6.20±0.09	0.264
Wing^2)^	2.13±0.16	2.32±0.05	2.17±0.05	2.04±0.07	0.255
Abdominal fat^2)^	3.10±0.16	3.24±0.02	3.43±0.14	3.32±0.08	0.259

1) Control: without sunflower meal and multienzymes; soybean meal was replaced with sunflower meal at levels of 3%, 6%, and 9% in 3SFM, 6SFM, and 9SFM, respectively, all diets were supplemented with 100 mg multienzymes per kg diet.

2) Percent of carcass weight.

**Table 5:** Effect of partially replacing soybean meal with sunflower meal with added multienzymes on internal organs indexes in broilers

Parameters (% of carcass weight)	Treatments^1)^				p-value

	Control	3SFM	6SFM	9SFM	
Heart	1.32±0.16	1.27±0.04	1.24±0.04	1.46±0.05	0.353
Liver	3.74±0.17	3.99±0.08	3.79±0.09	3.88±0.26	0.728
Gizzard	3.81±0.18	4.04±0.03	3.84±0.13	3.98±0.12	0.529
Bursa	0.45±0.01^b^	0.71±0.02^a^	0.66±0.02^a^	0.71±0.03^a^	0.001
Spleen	0.29±0.02	0.32±0.03	0.28±0.01	0.25±0.01	0.163
Thymus	0.77±0.05	0.86±0.02	0.75±0.03	0.89±0.06	0.093

1) Control: without sunflower meal and multienzymes; soybean meal was replaced with sunflower meal at levels of 3%, 6%, and 9% in 3SFM, 6SFM, and 9SFM, respectively, all diets were supplemented with 100 mg multienzymes per kg diet.

a,b Means within a row with different superscripts differ significantly (p<0.05).

**Table 6:** Effect of partially replacing soybean meal with sunflower meal with added multienzymes on intestinal segments in broilers

Parameters	Treatments^1)^				p-value

	Control	3SFM	6SFM	9SFM	
Weight (g)					
Duodenum	19.60±0.51^c^	25.00±0.32^a^	22.58±0.70^b^	20.60±0.37^bc^	0.001
Jejunum	32.18±2.41	35.20±0.37	33.50±1.32	30.60±2.11	0.323
Ileum	26.62±1.72	23.90±1.33	23.90±1.75	28.90±1.05	0.156
Ceca	11.82±0.26	12.26±0.25	11.40±0.75	11.50±0.45	0.609
Length (cm)					
Duodenum	14.68±0.26^b^	15.80±0.25^a^	14.90±0.19^ab^	14.87±0.33^ab^	0.036
Jejunum	63.08±0.35^bc^	77.30±0.37^a^	65.88±0.26^b^	62.70±0.28^c^	0.001
Ileum	63.12±3.64	63.50±2.91	63.50±3.47	64.68±1.74	0.985
Ceca	17.06±1.67	16.86±1.13	15.48±0.65	15.24±1.46	0.671
Jejunal morphology					
Villus height (μm)	1,187.60±25.70^a^	1,221.60±12.19^a^	1,151.80±27.12^a^	1,027.80±24.15^b^	0.001
Crypt depth (μm)	202.80±4.93^b^	241.80±4.82^a^	208.00±2.00^b^	207.00±2.00^b^	0.001
Villus/crypt	5.86±0.03^a^	5.06±0.06^b^	5.53±0.08^a^	4.97±0.13^b^	0.001

1) Control: without sunflower meal and multienzymes; soybean meal was replaced with sunflower meal at levels of 3%, 6%, and 9% in 3SFM, 6SFM, and 9SFM, respectively, all diets were supplemented with 100 mg multienzymes per kg diet.

a–c Means within a row with different superscripts differ significantly (p<0.05).

**Table 7:** Effect of partially replacing soybean meal with sunflower meal with added multienzymes on meat quality traits in broilers

Parameters	Treatments^1)^				p-value

	Control	3SFM	6SFM	9SFM	
Breast meat					
Shear force (g)	1,025.78±7.30	1,064.15±28.19	924.225±25.62	972.43±30.61	0.508
Cook loss (%)	26.38±1.36	27.89±1.48	26.84±1.09	26.31±1.48	0.833
WHC (%)	44.75±1.63	45.06±0.89	45.51±0.39	46.24±0.65	0.747
pH at 24 h	5.90±0.07^b^	6.10±0.02^a^	6.03±0.02^ab^	6.16±0.03^a^	0.006
L*	51.58±1.35	49.33±0.81	48.52±1.67	50.06±1.49	0.456
a*	10.03±1.71	7.78±0.52	9.19±1.21	7.79±0.87	0.457
b*	13.58±0.53^a^	10.57±0.26^b^	13.69±0.35^a^	10.41±0.94^b^	0.001
Thigh meat					
Cook loss (%)	31.91±0.53^ab^	35.62±0.98^a^	31.76±1.20^b^	34.51±0.94^ab^	0.024
WHC (%)	41.82±1.98	41.40±1.04	41.49±1.42	39.29±0.20	0.727
pH at 24 h	6.07±0.01^b^	6.44±0.03^a^	6.33±0.05^a^	6.35±0.06^a^	0.002
L*	53.37±2.32	53.48±2.11	52.74±2.84	48.71±2.41	0.474
a*	6.74±0.97	9.70±0.94	9.13±1.06	8.00±0.36	0.117
b*	7.65±1.21	10.48±1.17	9.82±1.77	10.16±1.27	0.477

WHC, water holding capacity.

1) Control: without sunflower meal and multienzymes; soybean meal was replaced with sunflower meal at levels of 3%, 6%, and 9% in 3SFM, 6SFM, and 9SFM, respectively, all diets were supplemented with 100 mg multienzymes per kg diet.

a,b Means within a row with different superscripts differ significantly (p<0.05).

**Table 8:** Effect of partially replacing soybean meal with sunflower meal with added multienzymes on ileal digestibility of nutrients in broilers

Parameters (%)	Treatments^1)^				p-value

	Control	3SFM	6SFM	9SFM	
Dry matter	78.76±0.44	81.04±1.28	81.27±1.23	81.53±0.81	0.695
Crude protein	69.46±0.08^b^	72.35±0.25^a^	71.79±0.38^a^	70.54±0.22^b^	0.001
Ether extract	74.19±0.50	75.11±1.31	72.16±0.84	72.53±1.90	0.356
Crude fiber	15.19±0.03	17.99±0.39	16.53±0.17	16.82±0.32	0.091

1) Control: without sunflower meal and multienzymes; soybean meal was replaced with sunflower meal at levels of 3%, 6%, and 9% in 3SFM, 6SFM, and 9SFM, respectively, all diets were supplemented with 100 mg multienzymes per kg diet.

a,b Means within a row with different superscripts differ significantly (p<0.05).

**Table 9:** Effect of partially replacing soybean meal with sunflower meal with added multienzymes on digestive enzymes activities in broilers

Parameters (U/mg)	Treatments^1)^				p-value

	Control	3SFM	6SFM	9SFM	
Lipase	22.38±0.66	23.43±1.43	22.46±1.59	22.75±2.06	0.960
Amylase	13.53±0.95^b^	16.87±0.64^a^	16.30±0.71^ab^	15.64±0.80^ab^	0.041
Protease	76.36±0.58^b^	85.18±0.53^a^	78.20±0.30^b^	78.01±0.35^b^	0.001

1) Control: without sunflower meal and multienzymes; soybean meal was replaced with sunflower meal at levels of 3, 6 and 9% in 3SFM, 6SFM, and 9SFM, respectively, all diets were supplemented with 100 mg multienzymes per kg diet.

a,b Means within a row with different superscripts differ significantly (p<0.05).

**Table 10:** Effect of partially replacing soybean meal with sunflower meal with added multienzymes on cecal microbiota in broilers

Parameters (logCFU/g)	Treatments^1)^				p-value

	Control	3SFM	6SFM	9SFM	
*Lactobacillus* spp.	8.40±0.24	10.40±1.21	11.00±1.41	10.20±1.77	0.537
*Bifidobacterium* spp.	7.40±0.24	9.60±1.54	9.80±1.59	9.80±1.39	0.515
*Clostridium perfringen*	6.40±0.68	5.20±0.92	5.00±1.14	5.00±1.30	0.740
*Escherichia coli*	6.60±1.03	5.60±1.12	4.40±1.36	4.40±1.33	0.532

1) Control: without sunflower meal and multienzymes; soybean meal was replaced with sunflower meal at levels of 3%, 6%, and 9% in 3SFM, 6SFM, and 9SFM, respectively, all diets were supplemented with 100 mg multienzymes per kg diet.
